# The associations of maternal liver biomarkers in early pregnancy with the risk of gestational diabetes mellitus: a prospective cohort study and Mendelian randomization analysis

**DOI:** 10.3389/fendo.2024.1396347

**Published:** 2024-05-21

**Authors:** Hui Liu, Libi Zhang, Haoyue Cheng, Peihan Chi, Yan Zhuang, Xialidan Alifu, Haibo Zhou, Yiwen Qiu, Ye Huang, Diliyaer Ainiwan, Shuting Si, Yunxian Yu

**Affiliations:** ^1^ Clinical Research Center, Sir Run Run Shaw Hospital, School of Medicine, Zhejiang University, Hangzhou, China; ^2^ Department of Public Health and Department of Anesthesiology, Second Affiliated Hospital of Zhejiang University School of Medicine, Hangzhou, China; ^3^ Department of Epidemiology & Health Statistics, School of Public Health, School of Medicine, Zhejiang University, Hangzhou, China; ^4^ Yiwu Maternity and Children Hospital (Yiwu Branch of Children’s Hospital Zhejiang University School of Medicine), Yiwu, China

**Keywords:** liver biomarkers, gestational diabetes mellitus, prospective cohort study, Mendelian randomization, interaction

## Abstract

**Background:**

Associations of liver function with the risk of gestational diabetes mellitus (GDM) remain unclear. This study aimed to examine the relationship and the potential causality between maternal liver biomarkers and the risk of subsequent GDM, as well as to evaluate the interaction between liver biomarkers and lipids on GDM risk.

**Methods:**

In an ongoing Zhoushan Pregnant Women Cohort, pregnant women who finished the first prenatal follow-up record, underwent liver function tests in early pregnancy, and completed the GDM screening were included in this study. Logistic regression models were used to investigate the association, and the inverse-variance weighted method supplemented with other methods of two-sample Mendelian randomization (MR) analysis was applied to deduce the causality.

**Results:**

Among 9,148 pregnant women, 1,668 (18.2%) developed GDM. In general, the highest quartile of liver function index (LFI), including ALT, AST, GGT, ALP, and hepatic steatosis index, was significantly associated with an increased risk of GDM (OR ranging from 1.29 to 3.15), especially an elevated risk of abnormal postprandial blood glucose level. Moreover, the causal link between ALT and GDM was confirmed by the MR analysis (OR=1.28, 95%CI:1.05-1.54). A significant interaction between AST/ALT and TG on GDM risk was observed (*P*_interaction_ = 0.026).

**Conclusion:**

Elevated levels of LFI in early pregnancy were remarkably associated with an increased risk of GDM in our prospective cohort. Besides, a positive causal link between ALT and GDM was suggested.

## 1 Introduction

Gestational diabetes mellitus (GDM) is impaired glucose tolerance of varying degrees first identified during pregnancy (1), which poses both short-term health risks including dystocia and macrosomia, and long-term health risks including type 2 diabetes and cardiovascular disease for mothers and their offspring (2, 3). Additionally, the prevalence of GDM in China was high, and a meta-analysis reported that the median prevalence was 14.8% (ranging from 5.12% to 33.30%) (4). The pathogenesis of GDM is mainly related to β-cell dysfunction and insulin resistance (5); however, metabolic changes during pregnancy cannot be ignored.

Non-alcoholic fatty liver disease (NAFLD) is a core component of metabolic syndrome, which is characterized by hepatic steatosis with insulin resistance (6). Previous investigations found NAFLD in the first trimester was a risk factor for GDM (7), which is often accompanied by abnormally elevated liver enzymes, including alanine aminotransferase (ALT), aspartate aminotransferase (AST), gamma-glutamyl transferase (GGT), or alkaline phosphatase (ALP) (8). Although elevated liver enzymes might positively correlate with insulin resistance (9), it was controversial whether it would be a potential risk factor for GDM. A new meta-analysis found a robust significant association between GGT and GDM by pooling five studies, while a null association between ALT and GDM by pooling another five articles (10). However, there were sparse studies on the association of AST and ALP with the risk of GDM (10). A most recent prospective cohort study including 6,860 pregnant women showed that higher levels of liver function index (LFI) in early pregnancy were positively associated with the risk of GDM (11). However, the prevalence of GDM in that study was low (about 7.2%), and only four liver enzymes including ALT, AST, GGT, and ALP were considered. Besides, whether there is a causal link between liver enzymes and GDM risk has yet to be clarified. Unlike conventional observational study design, which might be susceptible to reverse causation, Mendelian randomization (MR) analysis uses an instrumental variable (IV) to make a causal inference by assuming that single nucleotide polymorphisms (SNPs) are randomly allotted before disease onset (12).

In our prospective pregnancy cohort study, we aim to examine the association between maternal LFI in early pregnancy and the risk of subsequent GDM and its subtypes. A causal link between liver enzymes and GDM risk is confirmed by a two-sample MR approach. Considering the effect of lipids on the progress of GDM, the interaction of liver enzymes and lipids on GDM risk is further evaluated.

## 2 Materials and methods

### 2.1 Study design and population

Zhoushan Pregnant Women Cohort (ZPWC) is an ongoing prospective cohort that recruits pregnant women over 18 years old at 8-14 weeks of gestation. Detailed information on ZPWC was described in the previous article (13). All data in this study was obtained from a structured questionnaire and a comprehensive Electronic Medical Record System (EMRS) in Zhoushan Maternal and Child Care Hospital, Zhejiang. The work was conducted according to the Declaration of Helsinki and has been approved by the Institutional Review Board of Zhoushan Maternal and Child Health Care Hospital (Ethical Approval Code: 2011-05). Informed consent was obtained from all subjects involved in this study.

Until July 2022, a total of 9,589 pregnant women who had complete records of the first prenatal follow-up were recruited in this cohort. Of these, we excluded participants with missing data on liver enzyme tests in early pregnancy and GDM evaluation. Pregnant women with a history of hypertensive disorders, diabetes mellitus, heart disease, hepatobiliary disease, or kidney disease were further excluded. Lastly, participants with abnormally elevated liver enzymes (ALT > 90 U/L, AST > 80 U/L, GGT > 90 U/L, and ALP > 240 U/L) were ruled out.

### 2.2 Exposures

We obtained the liver function tests of pregnant women from the biochemical examination database. Venous blood was collected at 8-14 gestational weeks, and serum liver enzymes including ALT, AST, GGT, and ALP were quickly measured by a Hitachi 7600 automatic biochemical analyzer. As a noninvasive screening tool for NAFLD, hepatic steatosis index (HSI) was calculated according to the following formula: HSI=8 × ALT(U/L) ÷ AST(U/L) + BMI + 2(female) (14). Blood lipid biomarkers including total cholesterol (TC), triglycerides (TG), low-density lipoprotein cholesterol (LDL-C), and high-density lipoprotein cholesterol (HDL-C) were also extracted from biochemical databases.

### 2.3 Definition of outcome

Pregnant women without apparent diabetes routinely undergo a 75g OGTT after an overnight fast at 24 to 28 weeks of gestation. GDM was diagnosed if any OGTT plasma glucose values were at or above the following critical values: fasting blood glucose (FBG) ≥ 5.1 mmol/L, 1-h post-load blood glucose (1-h PBG) ≥ 10 mmol/L, and 2-h post-load blood glucose (2-h PBG) ≥ 8.5 mmol/L. Moreover, GDM was divided into three subtypes: isolated impaired fasting glucose (i-IFG) was an individual with an elevated level of FBG, isolated impaired glucose tolerance (i-IGT) was an individual with an elevated level of PBG, and b-GDM was an individual with elevated levels of both FBG and PBG (15).

### 2.4 Data sources for MR analysis

Summary statistics of IV-exposure (ALT, AST, GGT, and ALP) were extracted from the latest genome-wide association study (GWAS) including 500,000 European participants of UK Biobank (UKBB) (16). There are three basic assumptions including the relevance assumption, the independence assumption, and the exclusion-restriction assumption needing to be satisfied when selecting IVs. Firstly, biallelic SNPs with *P* value reaching genome-wide significance (*P* < 5.0 × 10^−8^) and minor allele frequency greater than 0.01 were selected. Then, a clumping algorithm with the parameter setting as r^2 ^= 0.001 and kb = 10,000 was used to eliminate the linkage disequilibrium among selected SNPs. Last, palindromic SNPs or pleiotropic SNPs associated with other traits (like BMI and lipids) other than exposures and outcomes according to the GWAS Catalog (accessed on Jan 2024) were excluded. Summary statistics of GDM were extracted from the FinnGen cohort (17), which included 5,687 GDM cases and 117,892 controls of the European population. The F-statistics of ALT, AST, GGT, and ALP were 79.4, 113.8, 152.9, and 100.3, respectively, indicating strong IVs (F-statistics > 10) for our exposures of interest (18).

### 2.5 Statistical analysis

Continuous variables with a normal distribution were presented as mean ± standard deviation (SD) and compared by independent t-tests. Non-normally distributed continuous variables were presented as median and interquartile range (IQR) and compared by the Mann-Whitney test. Categorical parameters were described as number (N) and percentage (%). Disordered classification data were compared using the Chi-square test., while rank data were compared using the Mann-Whitney test. LFI were divided into four categories based on their quartiles. A logistic regression model was used to assess the association of LFI in early pregnancy and the risk of GDM. Covariates were selected based on previous investigations (19), which included maternal age, BMI in the first trimester, educational level, gravidity, parity, weight gain before OGTT, gestational age of measurement, smoking status, and drinking consumption. Because BMI was included in the calculation of HSI, BMI was treated as a categorical variable (< 18.5, 18.5-23.9, 24.0-27.9, and ≥ 28) other than a continuous variable when testing the association between HSI and GDM. We performed tests for linear trend by assigning the median value of each category of LFI as a continuous variable in the models. Restricted cubic spline (RCS) functions with 4 knots were applied to examine whether there was a non-linear relationship between liver biomarkers and the blood glucose values. A linear regression model was used if the relationships were linear, and a piecewise regression model was performed if the relationships were non-linear. A multinomial logistic regression model was applied to assess the association of liver biomarkers in early pregnancy and the risk of GDM subtypes. To evaluate whether the relationship between liver biomarkers and GDM was independent of BMI, we performed the sensitivity analysis stratified by overweight (BMI ≥ 24 kg/m^2^). To validate the robustness of the results, we performed a series of sensitivity analyses by excluding individuals with liver enzyme levels exceeding the clinical reference range (ALT > 45 U/L, AST > 40 U/L, GGT > 45U/L, ALP > 120 U/L) (n=352). In addition, we performed crossover analysis to assess the joint effects and interaction between LFI and lipids on the risk of GDM. All statistical analyses were performed in R software (version 4.2.1), and *P* < 0.05 was considered statistically significant.

A two-sample MR analysis was conducted to evaluate the potential causal relationship between liver enzymes and GDM risk using the TwoSampleMR package in R software. The inverse-variance weighted (IVW) approach, calculated as a ratio of IV-outcome association to IV-exposure association for each IV, then combined multiple IVs weighted by the reciprocal of the outcome variance, was regarded as the primary method to estimate the causality (20). Cochran’s Q test was used to assess the heterogeneity. In order to rule out the effect of potential horizontal pleiotropy, other MR methods including. the weighted median method (21), MR-Egger regression method (22), and MR-pleiotropy residual sum and outlier (MR-PRESSO) method (23) were employed to complement the IVW approach. Moreover, considering that BMI is closely related to liver enzymes, summary statistics for BMI estimated from the European population of the UKBB database were used in multivariable MR (MVMR) analysis, which provided estimates of the causal link between liver enzymes and the risk of GDM independent of BMI (24).

## 3 Results

### 3.1 Baseline characteristics of the participants

As shown in **Supplementary Figure 1**, 9,148 pregnant women were included in this study. Of those, 1,668 (18.2%) pregnant women with GDM were identified (**Table 1**). Women in the GDM group were older, had a higher BMI in early pregnancy, lower levels of education, less weight gain before OGTT, and had a higher proportion of both gravidity and parity compared with pregnant women in the non-GDM group. However, smoking status, and alcohol consumption between the two groups were well balanced. In addition, women in the GDM group had significantly higher levels of ALT, AST, GGT, ALP, and HSI, and a lower level of AST/ALT.

**Table 1:** Comparison of maternal characteristics.

Variables	GDM (N=1668)	Non-GDM (N=7480)	*P*
Median (IQR)			
Age, years,	30.0 (27.0, 34.0)	28.0 (26.0, 31.0)	<0.001
BMI, kg/m^2^	21.9 (19.9, 24.5)	20.8 (19.2, 22.8)	<0.001
Weight gain before OGTT, kg	10.0 (7.5, 12.5)	12.3 (9.7, 14.9)	<0.001
ALT, U/L	14.0 (11.0, 21.0)	13.0 (10.0, 19.0)	<0.001
AST, U/L	17.0 (15.0, 20.0)	17.0 (14.0, 19.0)	<0.001
GGT, U/L	12.0 (10.0, 17.0)	11.0 (9.0, 15.0)	<0.001
ALP, U/L	51.0 (44.0, 59.0)	49.0 (43.0, 57.0)	<0.001
AST/ALT	1.1 (0.9, 1.4)	1.2 (0.9, 1.5)	<0.001
HSI	31.3 (28.3, 35.4)	29.7 (27.3, 32.8)	<0.001
N (%)			
Education			
Primary or below	305 (18.3)	1243 (16.6)	0.027
Middle	262 (15.7)	1138 (15.2)	
High	521 (31.2)	2303 (30.8)	
College or above	580 (34.8)	2796 (37.4)	
Gravidity			
1	684 (41.0)	3373 (45.1)	0.003
≥2	984 (59.0)	4107 (54.9)	
Parity			
0	1060 (63.5)	5185 (69.3)	<0.001
≥1	608 (36.5)	2295 (30.7)	
Smoking status			
No	1637 (98.1)	7352 (98.3)	0.695
Yes	22 (1.3)	82 (1.1)	
Unknown	9 (0.5)	46 (0.6)	
Alcohol consumption			
No	1639 (98.3)	7325 (97.9)	0.481
Yes	17 (1.0)	104 (1.4)	
Unknown	12 (0.7)	51 (0.7)	

GDM, gestational diabetes mellitus; BMI, body mass index; OGTT, oral glucose tolerance tests; ALT, alanine aminotransferase; AST, aspartate aminotransferase; GGT, gamma-glutamyl transferase; ALP, alkaline phosphatase; HSI, hepatic steatosis index.

### 3.2 Associations of maternal liver biomarkers in early pregnancy with GDM

**Figure 1:**
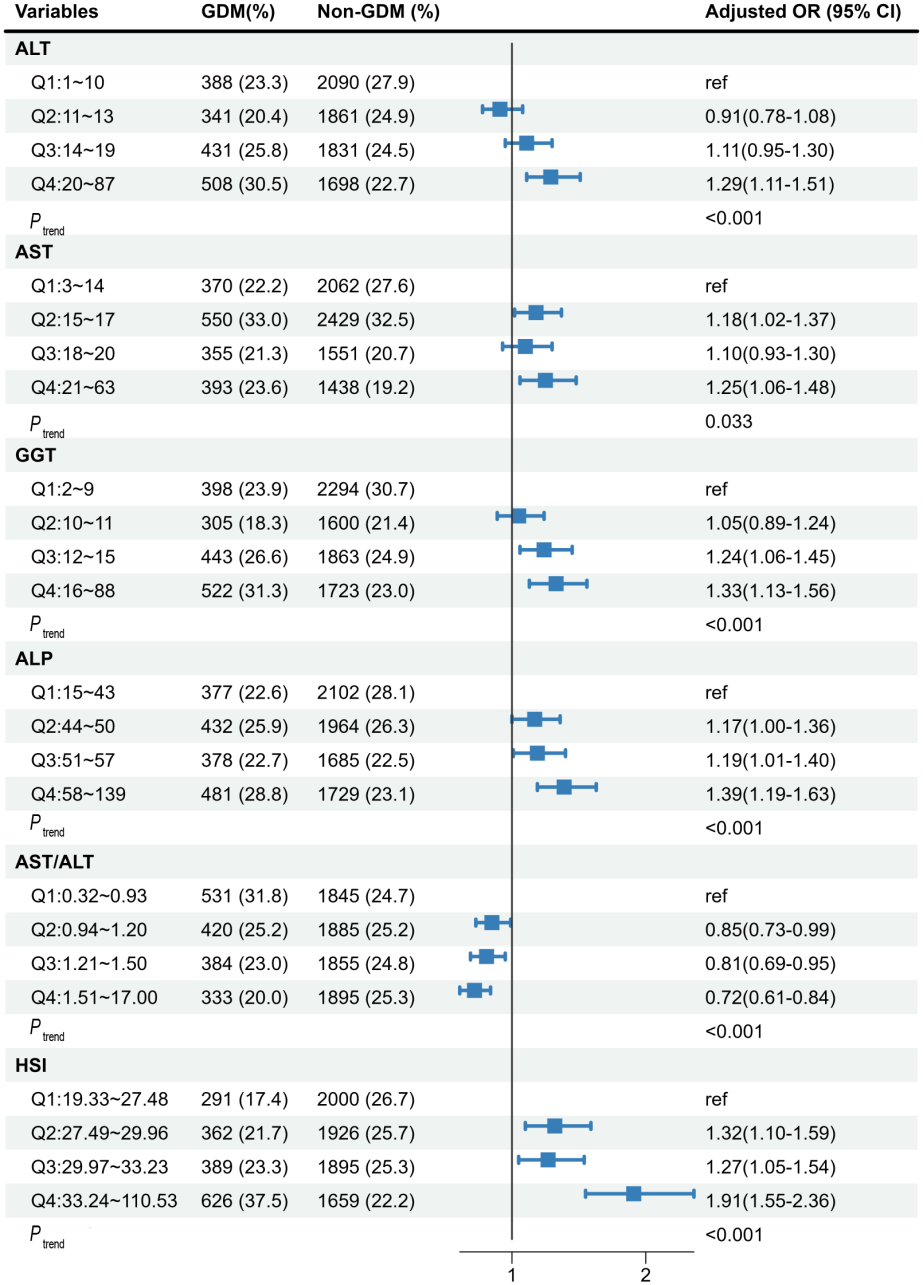
The associations of maternal liver biomarkers in early pregnancy with risk of GDM. The model was adjusted for age, maternal BMI in the first trimester, educational level, gravidity, parity, weight gain before OGTT, gestational age of measurement, smoking, and alcohol consumption. GDM, gestational diabetes mellitus; ALT, alanine aminotransferase; AST, aspartate aminotransferase; GGT, gamma-glutamyl transferase; ALP, alkaline phosphatase; HSI, hepatic steatosis index.

**Figure 1** shows the associations of maternal liver biomarkers in early pregnancy with the risk of GDM. We observed significant associations of higher levels of all LFI (Q4 vs. Q1) with the risk of GDM (*P*_trend_ < 0.05). After adjusting for covariates, pregnant women in the top quartile of ALT, AST, GGT, ALP, and HSI was respectively associated with a 1.29-fold (95%Cl: 1.11-1.51), 1.25-fold (95%Cl: 1.06-1.48), 1.33-fold (95%Cl: 1.13-1.56), 1.39-fold (95%Cl: 1.19-1.63), and 1.91-fold (95%Cl: 1.55-2.36) increased risk of GDM compared with those in the bottom quartile. Moreover, women with AST/ALT in the top quartile had a decreased risk of GDM (OR: 0.72, 95%Cl: 0.61-0.84).

### 3.3 Causal link between maternal liver biomarkers and GDM

**Figure 2:**
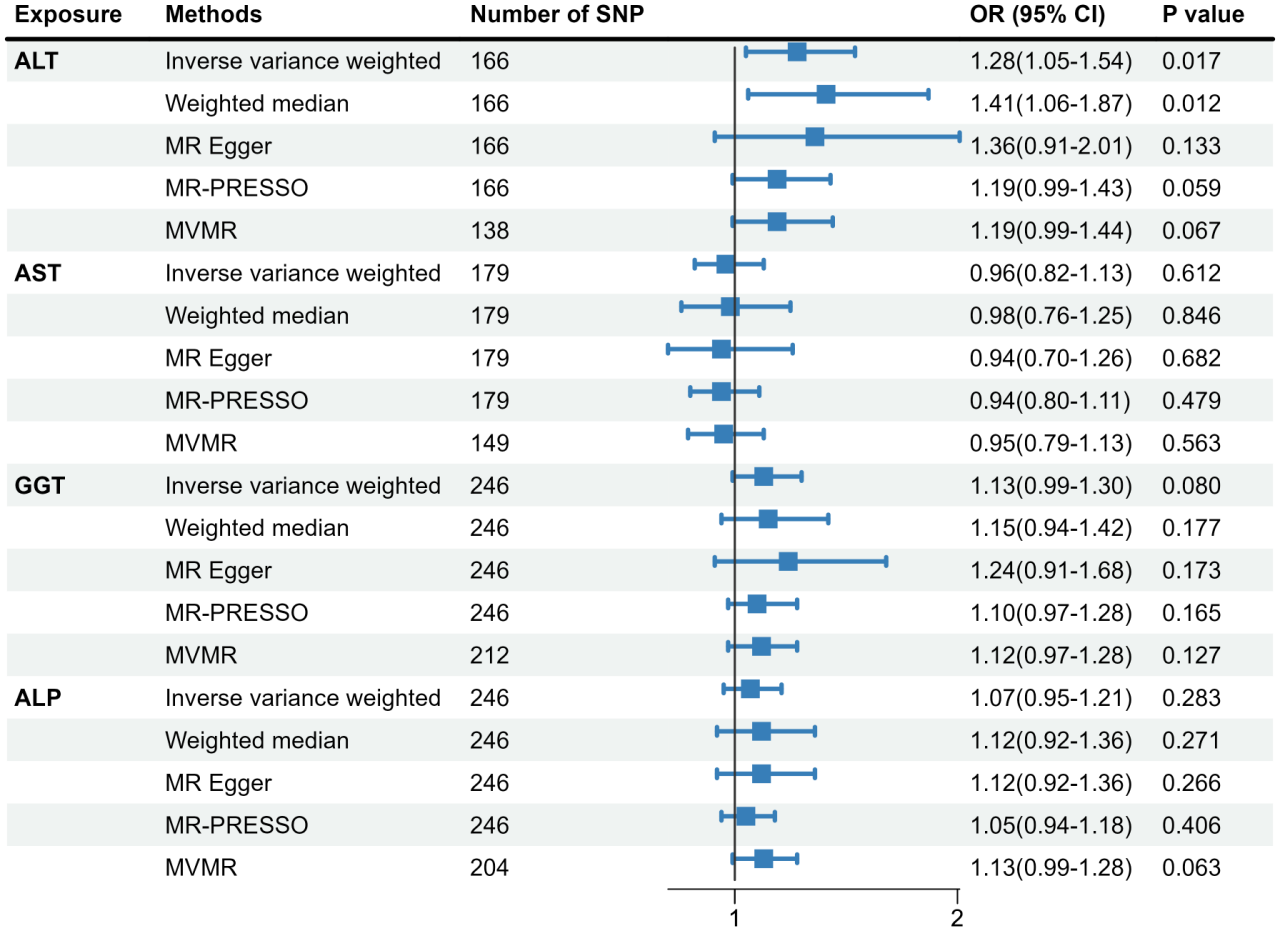
Causal association of genetically predicted liver biomarkers with the risk of GDM. MR, Mendelian randomization; SNP, single nucleotide polymorphism; MR-PRESSO, MR-pleiotropy residual sum and outlier; MVMR, multivariable MR; GDM, gestational diabetes mellitus; ALT, alanine aminotransferase; AST, aspartate aminotransferase; GGT, gamma-glutamyl transferase; ALP, alkaline phosphatase.

As shown in **Supplementary Figure 2**, 166 SNPs associated with ALT, 179 SNPs associated with AST, 246 SNPs associated with GGT, and 246 SNPs associated with ALP were included in the two-sample MR analysis. Detailed information on genetic variations was displayed in **Supplementary Tables 1**–**4**. **Figure 2** presents the causal link between liver enzymes and the risk of GDM. The estimate of the IVW method indicated that genetically predicted ALT was significantly associated with GDM risk (OR=1.28, 95%CI:1.05-1.54). The weighted median method confirmed the causality (OR=1.41, 95%CI:1.06-1.87), while the MR-Egger regression method yielded a similar but attenuated effect size (OR=1.36, 95%CI:0.91-2.01). Yet there was no evidence for causal associations of AST, GGT, and ALP with the risk of GDM. Considering the effect of BMI on the exposure-outcome association, the MVMR showed the same direction of the causal effect of ALT on GDM with the primary MR results (OR=1.19, 95%CI: 0.99-1.44), yet not reaching statistical significance. In addition, a certain level of heterogeneity was indicated on account of *P* < 0.05 by the IVW approach, but no horizontal pleiotropy was observed due to *P* > 0.05 according to the MR-Egger regression method and *P* > 1.0×10^-6^ according to the MR-PRESSO approach.

### 3.4 Associations of maternal liver biomarkers in early pregnancy with GDM subtypes

**Figure 3:**
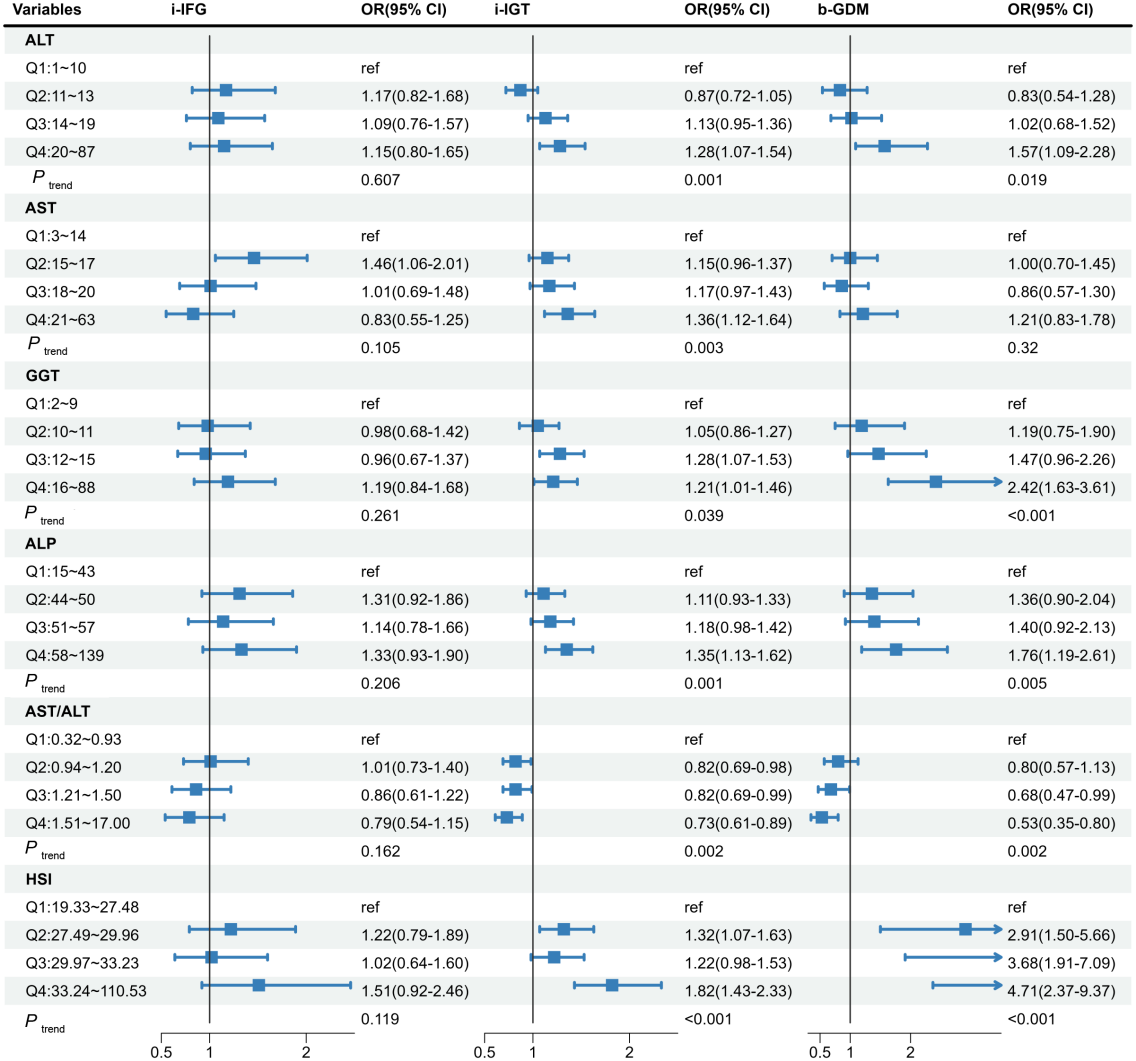
The associations of maternal liver biomarkers in early pregnancy with risk of the GDM subtypes. The model was adjusted for age, maternal BMI in the first trimester, educational level, gravidity, parity, weight gain before OGTT, gestational age of measurement, smoking, and alcohol consumption. GDM, gestational diabetes mellitus; ALT, alanine aminotransferase; AST, aspartate aminotransferase; GGT, gamma-glutamyl transferase; ALP, alkaline phosphatase; HSI, hepatic steatosis index; i-IFG, isolated impaired fasting glucose; i-IGT, isolated impaired glucose tolerance; b-GDM, both impaired fasting glucose and impaired glucose tolerance.

Multivariable restricted cubic spline regression analyses showed linear associations between most liver biomarkers and GDM risk (**Supplementary Figures 3**–**8**), but a non-linear shape between ALT and 2-h PBG (*P*_non-linear_ =0.003; **Supplementary Figure 3**), and a J-shape relationships of AST/ALT ratio with 1-h PBG and 2-h PBG (*P*_non-linear_ =0.006 for 1-h PBG; *P*_non-linear_ =0.011 for 2-h PBG, respectively; **Supplementary Figure 7**). Notably, only GGT and ALP increased the level of FBG; however, all LFI were significantly associated with the levels of PBG (**Supplementary Table 5**). Considering the diverse effect of liver enzymes on fasting and postprandial blood glucose, **Figure 3** shows the association of maternal liver biomarkers in early pregnancy with the risk of GDM subtypes. Consisted with the results of blood glucose level, the higher levels of LFI (Q4 vs. Q1) were significantly associated with i-IGT and b-GDM, but showed no association with i-IFG.

### 3.5 Sensitivity analysis

**Figure 4:**
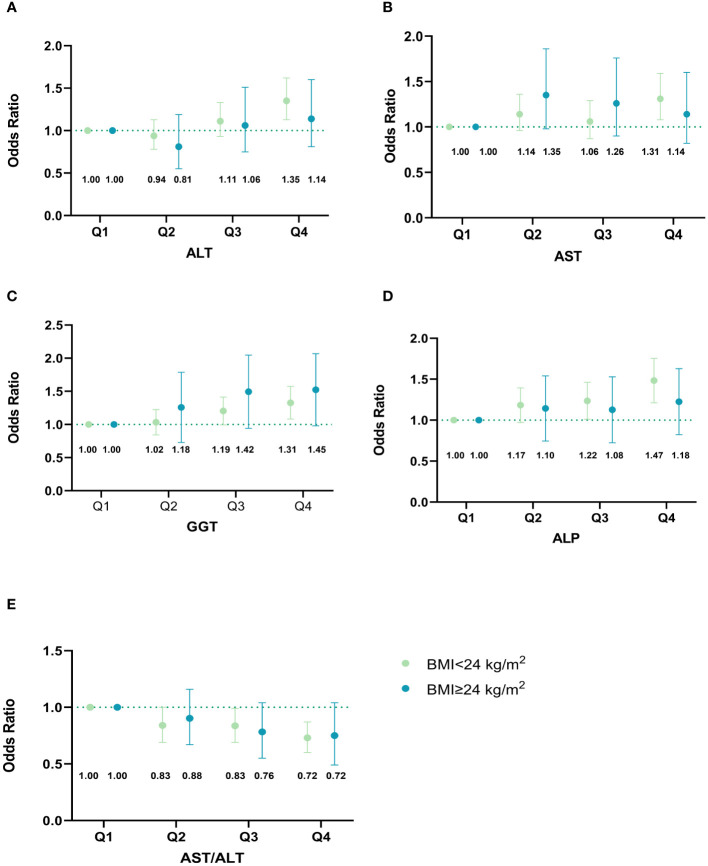
Associations of maternal liver biomarkers in early pregnancy with GDM risk stratified by overweight. **(A)** Associations of ALT in early pregnancy with GDM risk stratified by overweight; **(B)** Associations of AST in early pregnancy with GDM risk stratified by overweight; **(C)** Associations of GGT in early pregnancy with GDM risk stratified by overweight; **(D)** Associations of ALP in early pregnancy with GDM risk stratified by overweight; **(E)** Associations of AST/ALT ratio in early pregnancy with GDM risk stratified by overweight. The model was adjusted for age, maternal BMI in the first trimester, educational level, gravidity, parity, weight gain before OGTT, gestational age of measurement, smoking, and alcohol consumption. Based on their quartiles, live enzymes were divided into four categories (Q1-Q4). ALT was divided into 1~10,11~13,14~19, and 20~87 U/L. AST was divided into 3~14,15~17,18~20, and 21~63 U/L. GGT was divided into 2~9,10~11,12~15, and 16~88 U/L. ALP was divided into 15~43,44~50,51~57, and 58~139 U/L. AST/ALT ratio was divided into 0.32~0.93,0.94~1.20,1.21~1.50, and 1.51~17.00. The points in the figure represent the odds ratios (OR), and the error line represents the 95% confidence interval (CI). The numbers in the figure represent the corresponding OR value. GDM, gestational diabetes mellitus; ALT, alanine aminotransferase; AST, aspartate aminotransferase; GGT, gamma-glutamyl transferase; ALP, alkaline phosphatase.

**Figure 4** illustrates whether the effect of liver biomarkers on GDM risk is independent of BMI. Interestingly, the significant associations of all liver biomarkers with the risk of GDM were only observed in the non-overweight group. Additionally, we observed that the highest level of GGT was significantly associated with GDM in the overweight group. After excluding participants with liver enzyme levels exceeding the clinical reference range, the relationships between liver enzymes and the risk of GDM did not change (**Supplementary Table 6**). We also observed similar findings when the gestational age was restricted to the first trimester (8-12 weeks of gestation) (**Supplementary Table 7**). Due to missing data on lipid tests in early pregnancy, 4,340 pregnant women with both liver function and lipid tests were included in the crossover analysis. There were significant interactions between AST/ALT ratio and TG (*P _interaction_* = 0.026) (**Supplementary Table 8**). However, there was no significant interaction between other LFI and lipids in the development of GDM.

## 4 Discussion

In our ZPWC study, we found that elevated maternal LFI in early pregnancy was significantly associated with an increased risk of subsequent GDM. The relationships remained consistent only in non-overweight pregnant women. According to the two-sample MR analysis, we only observed a causal link between ALT and GDM risk. Furthermore, we found that elevated LFI in early pregnancy only raised the risk of postprandial glucose and its corresponding subtype of i-IGT and b-GDM. An interaction between AST/ALT and TG was noted in the development of GDM.

Our study illustrated an increased risk of GDM for pregnant women with elevated ALT in both the observational study and the MR study, while previous observational studies have shown inconsistent conclusions. A study of 17,359 pregnant women found that higher ALT levels in early pregnancy predicted an increased risk of GDM (25). However, a nested case-control study of 256 pregnant women with GDM showed no associations between ALT and GDM risk (26). Till now, no MR study has focused on the causal link between ALT and GDM risk. Instead, genetically predicted ALT was associated with a nearly 3-fold increased risk of type 2 diabetes according to an MR analysis performed by Liu et al. (27). Similar to our study, Wu et al. (28) found a positive association between AST and the risk of GDM. Whereas two other studies among pregnant women in the United States and China indicated null associations between AST and GDM risk (26, 29), which was in line with our findings of MR analysis. The possible reasons for the inconsistent conclusions may be the small sample size, different study designs, detection methods of liver biomarkers, varying diagnostic criteria for GDM, heterogeneity of the study population, and adjusting various covariants in models.

Previous investigations studying the associations of GGT and ALP with the risk of GDM indicated a consistent finding with ours (11, 15, 28). A prospective cohort study of 6,860 Chinese women found that high levels of GGT and ALP in early pregnancy were associated with a 1.53-fold and 1.42-fold increased risk of GDM, respectively (28). However, the findings of our MR analysis did not provide evidence of causal associations of GGT and ALP with the risk of GDM. One possible explanation for the inconsistent results between the MR analysis and the cohort study could be the heterogeneity of the population. Specifically, the Chinese population was recruited in the cohort study, whereas the European participants were included in the MR analysis. Only one study containing 94 GDM women found lower AST/ALT had a good predictive ability for the development of GDM, which was consistent with our findings (30). The positive association between HSI and GDM in our work was in line with Wu’s study, which found that HSI was significantly associated with a 1.12-fold risk of developing GDM even after adjustment for metabolic risk factors (31). Although the underlying mechanism between these biomarkers and GDM is unclear, hepatic lipid accumulation, insulin resistance, and oxidative stress may be involved in the development of GDM (32–34). More research into the mechanisms between liver function and GDM risk may be warranted.

Elevated liver enzyme levels increased the risk of GDM even within the normal clinical reference range, which has been confirmed by other studies (25, 35). It implied that pregnant women with higher levels of LFI might be at an increased risk of GDM. Unlike our study, which found the positive associations between LFI and GDM risk remained only in non-overweight women, a prospective cohort study of 6,211 pregnant women showed the association of elevated liver enzymes with GDM was independent of pre-pregnancy overweight (11). Limited power caused by the small sample size of overweight pregnant women in our study might explain the null association.

Furthermore, we tested whether liver biomarkers had different effects on GDM subtypes. Interestingly, our study showed that maternal elevated liver enzyme levels and HSI in early pregnancy increased the risk of i-IGT and b-GDM, but not i-IFG. The reason for this finding may be that postprandial plasma glucose level is primarily determined by the liver, which is responsible for handling the oral glucose load equivalent to approximately 60-65%, and any impairment of liver function may lead to postprandial hyperglycemia (36). Moreover, elevated liver enzyme levels are biomarkers of increased insulin resistance and decreased insulin sensitivity (37), which may lead to postprandial hyperglycemia. A study of 6,411 Finnish participants found that peripheral insulin resistance was more significant in patients with i-IGT than patients with i-IFG (38), which may explain that elevated liver enzymes are associated with PBG rather than FBG.

Because liver function and lipids both play an essential role in the occurrence of GDM, we further performed interaction tests of maternal liver biomarkers and lipids in early pregnancy on GDM risk. Our study found that low levels of AST/ALT ratio and high levels of TG had a significant interaction with an increased risk of GDM, while no interaction between other liver biomarkers and lipids on the risk of GDM. However, the interaction analysis results should be interpreted cautiously since the related mechanism is unclear. Additional studies with a large sample size are needed for definitive conclusions.

The strength of this study was integrating a prospective cohort study with a relatively large sample size and a two-sample MR study to explore the potential causal association between liver enzymes and GDM risk. Given that the causes of different subtypes of GDM may differ, the effects of liver biomarkers on GDM subtypes were also examined. Besides, the joint effect and the interaction test between liver enzymes and lipid levels on the risk of GDM were considered in our study. However, some limitations cannot be ruled out. Firstly, our study measured liver enzyme levels only once during the first trimester, which may introduce measurement errors. Secondly, data on liver fat content were unavailable, which may mediate the association between liver biomarkers and GDM risk. As a supplementary, a sensitive analysis stratified by BMI in early pregnancy was conducted to indicate the influence of body fat. Moreover, information on insulin, physical activity, and dietary habits is unavailable, which may affect the relationship between liver biomarkers and GDM risk. Lastly, due to the unavailability of genetic data for GDM in Asian populations, our MR analysis was performed in the European population. Thus, large GWAS studies are needed to identify genetic determinants of GDM in Asian women.

In conclusion, elevated LFI in early pregnancy was significantly associated with an increased risk of GDM, even within normal ranges. Yet, the MR analysis only confirmed a causal link between ALT and GDM risk. Further studies are warranted to validate the potential role of liver enzymes in early pregnancy in predicting GDM and its subtype, and to investigate the underlying biological mechanisms.

## Data availability statement

The original contributions presented in the study are publicly available. The datasets for this study can be found in the Figshare Digital Repository at dx.doi.org/10.6084/m9.figshare.25340728.

## Ethics statement

The studies involving humans were approved by Zhoushan Maternal and Child Health Care Hospital. The studies were conducted in accordance with the local legislation and institutional requirements. The participants provided their written informed consent to participate in this study.

## Author contributions

HL: Visualization, Methodology, Writing – original draft. LZ: Conceptualization, Writing – original draft. HC: Writing – review & editing, Data curation. PC: Writing – review & editing, Data curation. YZ: Writing – review & editing, Data curation. XA: Writing – review & editing, Investigation. HZ: Writing – review & editing, Investigation. YQ: Writing – review & editing, Investigation. YH: Writing – review & editing, Investigation. DA: Writing – review & editing, Investigation. SS: Writing – review & editing, Investigation. YY: Funding acquisition, Project administration, Resources, Supervision, Writing – review & editing.
