# Supplementary material for: The associations of maternal liver biomarkers in early pregnancy with the risk of gestational diabetes mellitus: a prospective cohort study and Mendelian randomization analysis
Source: Front Endocrinol (Lausanne). 2024 May 21;15:1396347. doi: 10.3389/fendo.2024.1396347 (PMC11148214; doi:10.3389/fendo.2024.1396347)
Supplement: Supplementary file 2 [file DataSheet_1.docx]

**Additional file 1**

**The Associations of Maternal Liver Biomarkers in Early Pregnancy with the Risk of Gestational Diabetes Mellitus: A Prospective Cohort Study and Mendelian Randomization Analysis**

**Figure S1.** Flowchart of the selection of the participants.

**Figure S2:** Flowchart of instrumental variables selection

**Figure S3:** Associations of ALT in early pregnancy with blood glucose level.

**Figure S4:** Associations of AST in early pregnancy with blood glucose level.

**Figure S5:** Associations of GGT in early pregnancy with blood glucose level.

**Figure S6:** Associations of ALP in early pregnancy with blood glucose level.

**Figure S7:** Associations of AST/ALT ratio in early pregnancy with blood glucose level.

**Figure S8:** Associations of HSI in early pregnancy with blood glucose level.


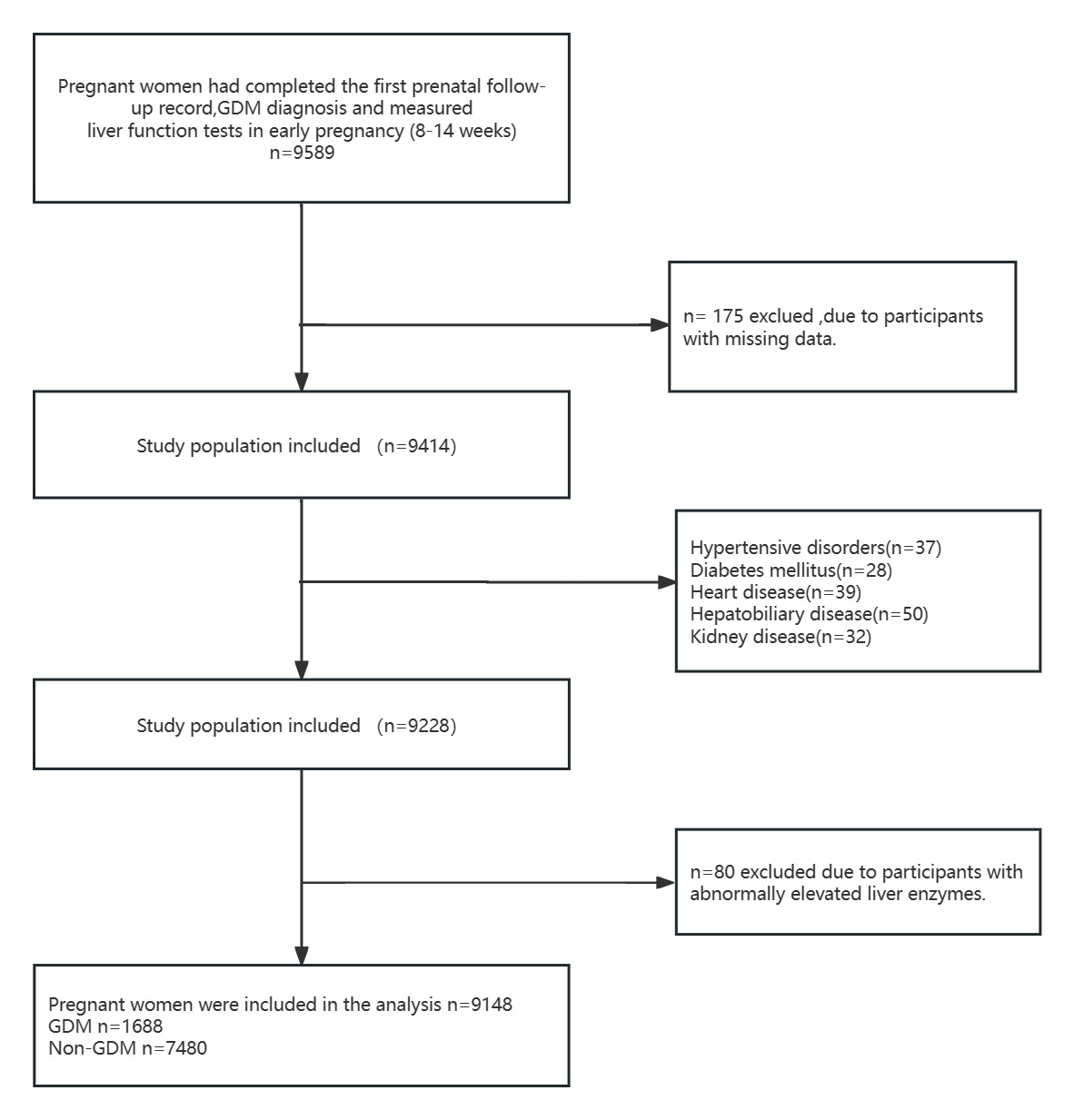


Figure S1: Flowchart of the selection of the participants.


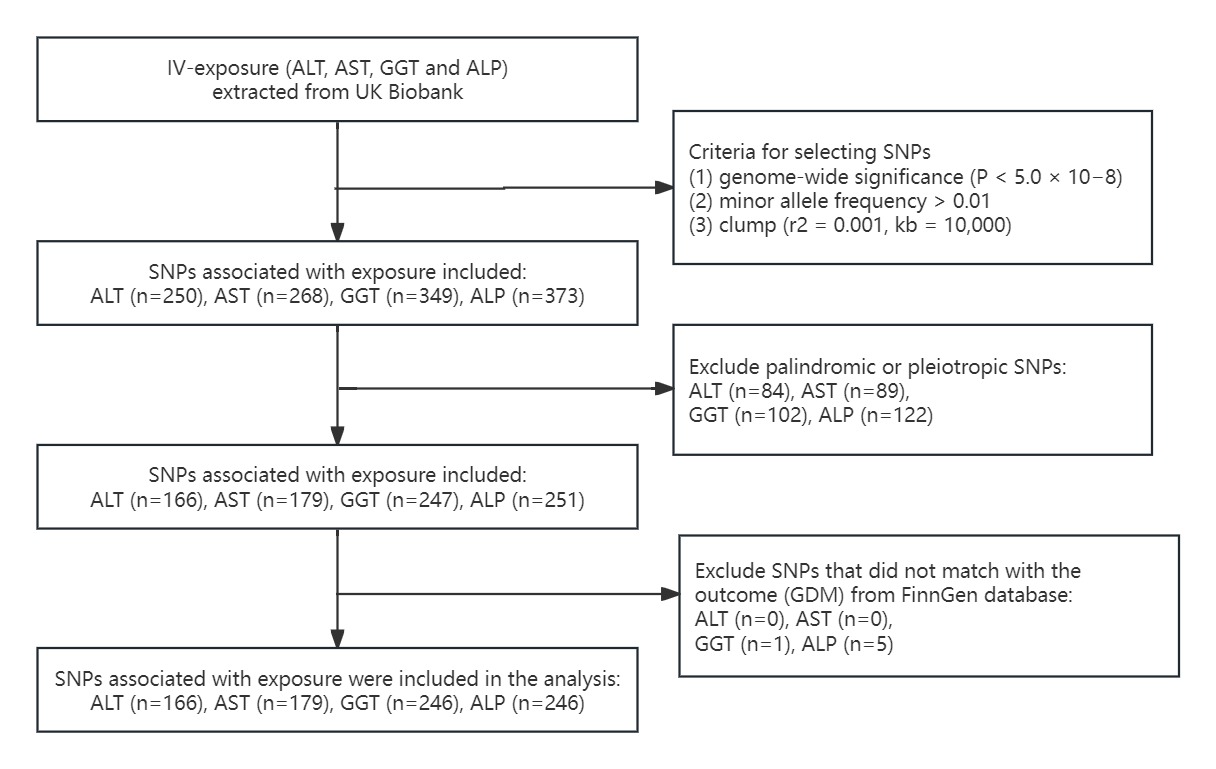


Figure S2: Flowchart of instrumental variables selection.

Figure S3: Associations of ALT in early pregnancy with blood glucose level. (A) Associations of ALT in early pregnancy with FBG. (B) Associations of ALT in early pregnancy with 1-h PBG. (C) Associations of ALT in early pregnancy with 2-h PBG.

^†^ The *P* values for non-linear associations were 0.181 (A), 0.177 (B), and 0.003 (C), respectively.

^‡^ The models were adjusted for age, maternal BMI in the first trimester, educational level, gravidity, parity, weight gain before OGTT, gestational age of measurement, smoking, and alcohol consumption.

Abbreviations: ALT: alanine aminotransferase; FBG: fasting blood glucose; PBG: post-load blood glucose.

Figure S4: Associations of AST in early pregnancy with blood glucose level. (A) Associations of AST in early pregnancy with FBG. (B) Associations of AST in early pregnancy with 1-h PBG. (C) Associations of AST in early pregnancy with 2-h PBG.

^†^ The *P* values for non-linear associations were 0.385 (A), 0.233 (B), and 0.139 (C), respectively. ^‡^ ^‡^ The models were adjusted for age, maternal BMI in the first trimester, educational level, gravidity, parity, weight gain before OGTT, gestational age of measurement, smoking, and alcohol consumption.

Abbreviations: AST: aspartate aminotransferase; FBG: fasting blood glucose; PBG: post-load blood glucose.

Figure S5: Associations of GGT in early pregnancy with blood glucose level. (A) Associations of GGT in early pregnancy with FBG. (B) Associations of GGT in early pregnancy with 1-h PBG. (C) Associations of GGT in early pregnancy with 2-h PBG.

^†^ The *P* values for non-linear associations were 0.552 (A), 0.139 (B), and 0.165 (C), respectively.

^‡^ The models were adjusted for age, maternal BMI in the first trimester, educational level, gravidity, parity, weight gain before OGTT, gestational age of measurement, smoking, and alcohol consumption.

Abbreviations: GGT: gamma-glutamyl transferase; FBG: fasting blood glucose; PBG: post-load blood glucose.

Figure S6: Associations of ALP in early pregnancy with blood glucose level. (A) Associations of ALP in early pregnancy with FBG. (B) Associations of ALP in early pregnancy with 1-h PBG. (C) Associations of ALP in early pregnancy with 2-h PBG.

^†^ The *P* values for non-linear associations were 0.107 (A), 0.942 (B), and 0.567 (C), respectively.

^‡^ The models were adjusted for age, maternal BMI in the first trimester, educational level, gravidity, parity, weight gain before OGTT, gestational age of measurement, smoking, and alcohol consumption.

Abbreviations: ALP: alkaline phosphatase; FBG: fasting blood glucose; PBG: post-load blood glucose.

Figure S7: Associations of AST/ALT in early pregnancy with blood glucose level. (A) Associations of AST/ALT in early pregnancy with FBG. (B) Associations of AST/ALT in early pregnancy with 1-h PBG. (C) Associations of AST/ALT in early pregnancy with 2-h PBG.

^†^ The *P* values for non-linear associations were 0.675 (A), 0.006 (B), and 0.011 (C), respectively.

^‡^ The models were adjusted for age, maternal BMI in the first trimester, educational level, gravidity, parity, weight gain before OGTT, gestational age of measurement, smoking, and alcohol consumption.

Abbreviations: ALT: alanine aminotransferase; AST: aspartate aminotransferase; FBG: fasting blood glucose; PBG: post-load blood glucose.

Figure S8: Associations of HSI in early pregnancy with blood glucose level. (A) Associations of HSI in early pregnancy with FBG. (B) Associations of HSI in early pregnancy with 1-h PBG. (C) Associations of HSI in early pregnancy with 2-h PBG.

^†^ The *P* values for non-linear associations were 0.630 (A), 0.278 (B), and 0.097 (C), respectively.

^‡^ The models were adjusted for age, maternal BMI in the first trimester, educational level, gravidity, parity, weight gain before OGTT, gestational age of measurement, smoking, and alcohol consumption. For HSI, BMI in the first trimester was treated as a categorical variable (<18.5, 18.5-23.9, 24.0-27.9 and ≥28.0 kg/m2) for adjustment in the modeling to avoid multicollinearity.

Abbreviations: HSI: hepatic steatosis index; FBG: fasting blood glucose; PBG: post-load blood glucose.
